# Targeted Suppression of CEACAM6 via pHLIP-Delivered RNAs in Pancreatic Ductal Adenocarcinoma

**DOI:** 10.3390/medicina61040598

**Published:** 2025-03-26

**Authors:** Hongsik Kim, Chang-Gok Woo, Seung-Myoung Son, Yong-Pyo Lee, Hee-Kyung Kim, Yaewon Yang, Jihyun Kwon, Ki-Hyeong Lee, Ho-Chang Lee, Ok-Jun Lee, Hye-Sook Han

**Affiliations:** 1 Department of Internal Medicine, Chungbuk National University Hospital, College of Medicine, Chungbuk National University, Cheongju 28644, Republic of Korea; kimhs5186@gmail.com (H.K.); knee49@naver.com (Y.-P.L.); heekyung.kim16@gmail.com (H.-K.K.); yaewonyang@gmail.com (Y.Y.); marioncrepe@gmail.com (J.K.); kihlee@chungbuk.ac.kr (K.-H.L.); 2 Department of Pathology, Chungbuk National University Hospital, College of Medicine, Chungbuk National University, Cheongju 28644, Republic of Korea; thewallflower@daum.net (C.-G.W.); da10na13@daum.net (S.-M.S.); fgump0@gmail.com (H.-C.L.); md5218@naver.com (O.-J.L.)

**Keywords:** pancreatic ductal adenocarcinoma, pHLIP, CEACAM6, drug delivery, tumor acidity

## Abstract

*Background and Objectives*: Carcinoembryonic antigen-related cell adhesion molecule 6 (CEACAM6) is involved in pancreatic cancer progression and is an attractive therapeutic target for pancreatic cancer. In this study, we evaluated the therapeutic efficacy of small-interfering RNA (siRNA) targeting CEACAM6 (siCEACAM6) and the CEACAM6-suppressive microRNA-29a (miR-29a) in a pancreatic ductal adenocarcinoma xenograft mouse model using pH-low insertion peptide (pHLIP) technology, which targets the acidic tumor microenvironment. *Materials and Methods*: The delivery vectors for siRNA and miRNA were constructed by conjugating the peptide nucleic acid forms of siCEACAM6 and miR-29a to a peptide with a pHLIP, enabling the transport of siRNA and miRNA across the plasma membrane. The tumor-suppressive effects of pHLIP-siCEACAM6 and pHLIP-miR-29a were assessed in vivo using a BALB/c xenograft mouse model with the injection of the CFPAC-1 human pancreatic ductal adenocarcinoma cell line. *Results*: The treatment of CFPAC-1 cells with pHLIP-siCEACAM6 and pHLIP-miR-29a under acidic pH conditions suppressed CEACAM6 expression and decreased cell viability. In a xenograft mouse model, the intravenous injection of pHLIP-siCEACAM6 and pHLIP-miR-29a suppressed tumor growth by up to 25.1% (*p* < 0.01) and 21.2% (*p* < 0.01), respectively, compared to the control mice treated with pHLIP-scr. *Conclusions*: Our results demonstrated the efficacy of the pHLIP-mediated delivery of siCEACAM6 and miR-29a as a promising therapeutic strategy in a pancreatic ductal adenocarcinoma xenograft mouse model. The pHLIP technology, which targets the acidic tumor microenvironment, represents an innovative approach to the delivery of small RNAs to pancreatic ductal adenocarcinoma cells, providing new potential strategies for pancreatic cancer treatment.

## 1. Introduction

Pancreatic ductal adenocarcinoma (PDAC) is a highly aggressive cancer with limited therapeutic options and poor survival outcomes [1], largely due to asymptomatic disease progression, diagnoses at advanced or metastatic stages, and resistance to standard systemic chemotherapy [2]. The global PDAC incidence is increasing at a steady pace; thus, the discovery of new therapeutic targets is essential for improving patient survival outcomes.

Several studies have highlighted carcinoembryonic antigen cell adhesion molecule 6 (CEACAM6) as a potential therapeutic target for various cancers [3]. Nearly 70% of epithelial malignancies, such as pancreatic, colon, non-small-cell lung, breast, and gastric cancers, overexpress CEACAM6 [4,5,6,7,8,9]. CEACAM6 is a glycoprotein anchored to the cell surface via glycosylphosphatidylinositol (GPI) and is a member of the carcinoembryonic antigen (CEA) family [10]. It is critically involved in cell adhesion, migration, and signal transduction, and its dysregulation is frequently observed in malignancies such as PDAC [10,11].

Multiple studies have reported that CEACAM6 is frequently overexpressed in PDAC and is associated with tumor progression, metastasis, and poor clinical outcomes [4,5,6,12]. CEACAM6 overexpression enhances pancreatic cancer-cell invasiveness and confers resistance to apoptosis and chemotherapy [13,14]. The mechanisms underlying this process involve the activation of the PI3K/Akt signaling pathway and disruption of cell–cell adhesion [15,16]. Preclinical studies have demonstrated the therapeutic potential of CEACAM6, showing that its downregulation suppresses tumor growth and increases cancer-cell sensitivity to chemotherapy [15,17,18]. These findings underscore the pivotal role of CEACAM6 in PDAC and suggest it as a potential therapeutic target.

pH-low insertion peptide (pHLIP) technology represents an innovative approach to targeted drug delivery, utilizing the acidic nature of the tumor microenvironment [19]. pHLIP has the unique ability to be selectively inserted into cell membranes under acidic conditions, which is a key characteristic of tumors. This pH-dependent insertion mechanism enables pHLIP to function as molecular carriers and efficiently deliver therapeutic molecules directly to tumor sites.

We previously identified CEACAM6 as a novel therapeutic target for non-small-cell lung cancer (NSCLC). Specifically, we demonstrated that microRNA-29a (miR-29a) functions as a tumor suppressor in NSCLC by regulating CEACAM6, suggesting that the miR-29a–CEACAM6 axis is a potential therapeutic target [20]. Additionally, we demonstrated the therapeutic efficacy of CEACAM6 gene silencing using a small-interfering RNA (siRNA) delivery platform in an acidic tumor microenvironment [21]. This strategy used pHLIP as a delivery vector for CEACAM6-targeting siRNA (siCEACAM6) and miR-29a in NSCLC cells.

In this study, we extended our previous findings by evaluating CEACAM6 as a novel therapeutic target for PDAC. By leveraging the high binding affinity of the peptide nucleic acid (PNA) forms of siCEACAM6 and miR-29a, we developed a pHLIP-fused delivery system with tumor-specific targeting properties and explored the therapeutic potential of CEACAM6 inhibition via siCEACAM6 and miR-29a in a PDAC xenograft model.

## 2. Materials and Methods

### 2.1. Synthesis of PNA-pHLIP

PNA-pHLIP was synthesized as previously described [20,21]. PNA oligomers, including siCEACAM6, miR-29a mimic, and scrambled siRNA, were obtained from PANAGENE (Daejeon, Republic of Korea). To construct the pHLIP-PNA, a pHLIP sequence was synthesized. Each PNA oligomer was conjugated to the C-terminal region of pHLIP through a disulfide bond, forming a stable conjugate.

### 2.2. Cell Culture

The human PDAC cell line CFPAC-1 (ATCC CRL-1918) was obtained from the American Type Culture Collection (ATCC, Manassas, VA, USA). CFPAC-1 cells were cultured in an RPMI complete culture medium, and cell cultures were maintained under a 5% CO_2_ atmosphere at 37 °C. For pH-regulated experiments, CFPAC-1 cells were cultured in complete a culture medium containing 10% fetal bovine serum (FBS), buffered to pH 7.4 using 4-(2-hydroxyethyl)-1-piperazineethanesulfonic acid (HEPES), or to pH 6.2 using 2-(N-morpholino)-ethanesulfonic acid (MES).

### 2.3. Transient Transfection and qRT-PCR for siCEACAM

The siRNA duplexes were acquired from Dharmacon Research, Inc. (Lafayette, CO, USA). A non-targeting siRNA was used as a negative control to verify the specificity of inhibition. The sequences of the siRNAs used in the CEACAM6 knockdown experiments included Seq #1, Seq #2, Seq #3, Seq #4, and a negative control, as previously described [21]. To assess CEACAM6 gene silencing, transfection of CFPAC-1 cells was performed with 10 nM siRNA duplexes using Lipofectamine RNAiMax (Thermo Fisher Scientific, Rockford, IL, USA). The degree of CEACAM6 silencing was determined via a quantitative real-time reverse transcription polymerase chain reaction (qRT-PCR) after 48 h.

For gene expression analysis, total RNA extraction was performed using TRIzol reagent (Invitrogen, Carlsbad, CA, USA), and complementary DNA (cDNA) synthesis was performed using the iScript™ cDNA synthesis kit (Bio-Rad, Hercules, CA, USA). qRT-PCR was performed using iQ SYBR Green Supermix (Bio-Rad). The S1000 thermal cycler real-time PCR system (Bio-Rad) was employed for thermal cycling and fluorescence detection. The qRT-PCR reactions were performed using the CFX96 Real-Time PCR System (Bio-Rad). Gene expression levels were analyzed relative to GAPDH expression levels.

### 2.4. Western Blot and Cell Proliferation Assay for pHLIP-miR-29a

To evaluate the effects of pHLIP-miR-29a on CEACAM6 inhibition and CFPAC-1 cell proliferation, Western blot analysis and cell proliferation assay were performed. CFPAC-1 cells were treated with pHLIP-PNA for 48 h before protein extraction. Then, proteins were extracted from the cells and separated. After blocking with 5% non-fat milk, membranes were incubated with either a mouse monoclonal anti-CEACAM6 antibody (9A6, Santa Cruz Biotechnology Inc., Dallas, TX, USA) or a mouse monoclonal anti-GAPDH antibody (6C5, Santa Cruz Biotechnology Inc., Dallas, TX, USA) as a loading control. Protein-complex detection was performed using enhanced chemiluminescence (ECL) reagents (Thermo Fisher Scientific).

A CFPAC-1 cell proliferation assay was performed using a Cell Proliferation Kit II (XTT) (Sigma-Aldrich, Inc., St. Louis, MO, USA). CFPAC-1 cells (5 × 10^4^/mL) were treated with pHLIP-miR-29a at varying concentrations (0, 100, 250, or 500 nM) in 96-well plates for 48 h. pHLIP-scr was used as a control and experiments were conducted under predefined pH conditions. After 48 h, cell proliferation was evaluated by adding 50 μL of XTT solution to each well, followed by a 2-h incubation at 37 °C. Absorbance was measured at 450 nm using a Lambda Bio-20 multiplate reader (Beckman Coulter, Brea, CA, USA). Cell proliferation was calculated as a percentage of the control group.

### 2.5. In Vivo Tumor Xenograft Experiments

Athymic BALB/c nude mice (5 weeks old, *n* = 5 mice per group) received subcutaneous injections of CFPAC-1 cells (9 × 10^6^ cells/mouse) into the right axilla. Tumor growth was assessed by measuring length (L), width (W), and height (H) using calipers, and the tumor volume was calculated using the formula: V = (L × W × H) × 0.5. At the end of the study, tumors were excised and weighed to determine the total weight. Once the tumors had formed (approximately 48.8 mm^3^), the mice were administered the pHLIP-PNA constructs intravenously.

### 2.6. Statistical Analysis

For animal experiments, mouse and tumor weights across groups were compared using Student’s *t*-test. Two-sided *p*-values were calculated, with statistical significance defined as *p* < 0.05. All statistical analyses were performed using GraphPad Prism 7 (GraphPad Software, La Jolla, CA, USA).

## 3. Results

### 3.1. Generation of pHLIP-siCEACAM6

**Figure 1:**
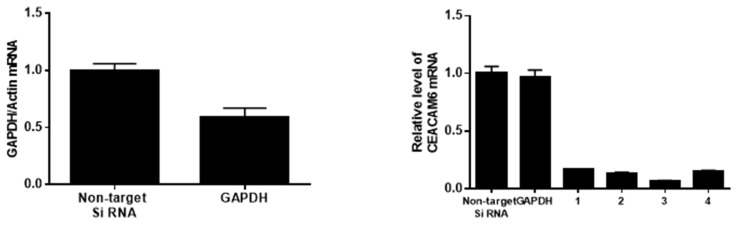
qRT-PCR analysis of CEACAM6 mRNA expression in CFPAC-1 cells transfected with non-target siRNA, GAPDH siRNA, or four different siCEACAM6 sequences.

For the generation of the pHLIP-siCEACAM6 construct, four different siRNA sequences were tested to evaluate their efficiency in CEACAM6 knockdown. The transfection of CFPAC-1 PDAC cells was performed using either a non-target siRNA, GAPDH siRNA, or four different siCEACAM6 sequences. After 48 h of transfection at a 10 nM concentration, all four siCEACAM6 sequences similarly suppressed CEACAM6 mRNA expression compared to the non-target siRNA (Figure 1). Based on this assessment, siCEACAM6 sequence #1 was selected for conjugation to pHLIP. The pHLIP-siCEACAM6 construct was synthesized by conjugating the C-terminal region of the PNA oligomer siCEACAM6 to pHLIP, generating a functional targeted delivery system.

### 3.2. CEACAM6 Inhibition and CFPAC-1 Cell Viability via pHLIP-miR-29a

**Figure 2:**
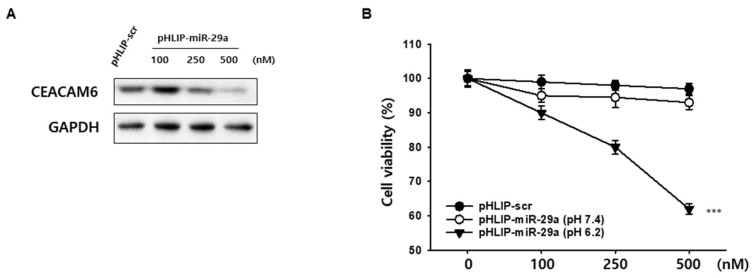
Activity of pHLIP-miR-29a in targeting CEACAM6 in CFPAC-1 cells. (**A**) Western blot analysis of CEACAM6 protein expression in CFPAC-1 cells treated with pHLIP-miR-29a at an acidic pH (6.2). (**B**) The effect of pHLIP-miR-29a on CFPAC-1 cell viability was evaluated under both neutral and acidic pH conditions. *** *p* < 0.001.

The pHLIP-miR-29a construct was synthesized by conjugating the C-terminal region of the PNA oligomer derived from the miR-29a mimic to pHLIP. A pHLIP-scr construct was also synthesized as a negative control. A single isomer of 5-carboxytetramethylrhodamine label was incorporated into the pHLIP-PNA oligomer. To investigate whether pHLIP-miR-29a effectively delivers miR-29a into cells and targets CEACAM6, CFPAC-1 PDAC cells were incubated at pH 6.2 for 48 h with varying concentrations (100, 250, or 500 nM) of pHLIP-miR-29a or pHLIP-scr. Western blot analysis revealed that pHLIP-miR-29a suppressed CEACAM6 expression in a dose-dependent manner at acidic pH, indicating the successful intracellular delivery of miR-29a and effective suppression of CEACAM6 (Figure 2A). Furthermore, pHLIP-mediated miR-29a delivery significantly reduced CFPAC-1 cell viability in a dose-dependent manner at an acidic pH, while no significant effect was observed at a neutral pH (Figure 2B).

### 3.3. Assessment of pHLIP-siCEACAM6 Therapeutic Efficacy in a Pancreatic Adenocarcinoma Xenograft Model

Next, we evaluated the antitumor efficacy of pHLIP-siCEACAM6 in vivo using a PDAC xenograft mouse model. BALB/c nude mice were subcutaneously injected with 9 × 10^6^ CFPAC-1 cells. Two weeks after the injection of 9 × 10^6^ CFPAC-1 cells, the mice were randomized into three groups and administered pHLIP-siCEACAM6 or pHLIP-scr (vector control) via tail-vein injection. To determine the most effective therapeutic dose, two groups of mice (*n* = 5 per group) received intravenous injections of pHLIP-siCEACAM6 at doses of 2 mg/kg and 4 mg/kg twice weekly for three weeks. After treatment, all mice were sacrificed for further analysis.

**Figure 3:**
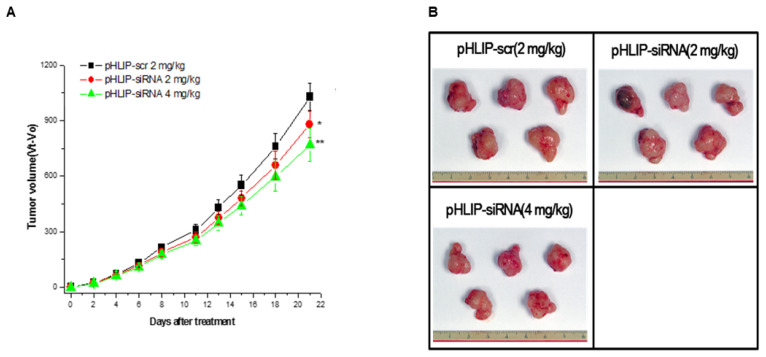
Administration of pHLIP-siCEACAM6 inhibits pancreatic tumor progression in a pancreatic ductal adenocarcinoma-bearing mouse model. (**A**) CFPAC-1 Tumor-bearing nude mice received intravenous injections of pHLIP-siCEACAM6, and tumor volumes were assessed at the indicated days following treatment (*n* = 5 mice per group). * *p* < 0.05; ** *p* < 0.01. (**B**) Representative tumor images were taken three weeks following administration of pHLIP-siCEACAM6 or pHLIP-scr at doses of 2 mg/kg or 4 mg/kg.

To evaluate the effects of pHLIP-siCEACAM6 on pancreatic tumor development, tumor growth was measured every 2–3 days throughout the study. Tumor volumes in pHLIP-siCEACAM6-treated mice were significantly smaller than those in pHLIP-scr-treated control mice (Figure 3, Supplementary Table S1). At a dose of 2 mg/kg, the tumor size in pHLIP-siCEACAM6-treated mice was reduced by 14.5% compared to those in pHLIP-scr-treated mice (*p* < 0.05). At a dose of 4 mg/kg, the tumor size was reduced by 25.1% (*p* < 0.01). Additionally, pHLIP-siCEACAM6-treated mice exhibited no changes in body weight or clinical signs of distress for toxicity assessment (Supplementary Figure S1A).

### 3.4. Assessment of pHLIP-miR-29a Therapeutic Efficacy in a Pancreatic Adenocarcinoma Xenograft Model

We further evaluated the antitumor efficacy of pHLIP-miR-29a in vivo using a PDAC xenograft mouse model. Two weeks after the injection of 9 × 10^6^ CFPAC-1 cells, the mice were randomized into three groups and administered pHLIP-miR-29a or pHLIP-scr (vector control) via tail vein injection. To determine the most effective therapeutic dose, two groups of mice (*n* = 5 per group) received intravenous injections of pHLIP-miR-29a at doses of 2 mg/kg and 4 mg/kg twice weekly for three weeks. After treatment, all mice were sacrificed for further analysis.

**Figure 4:**
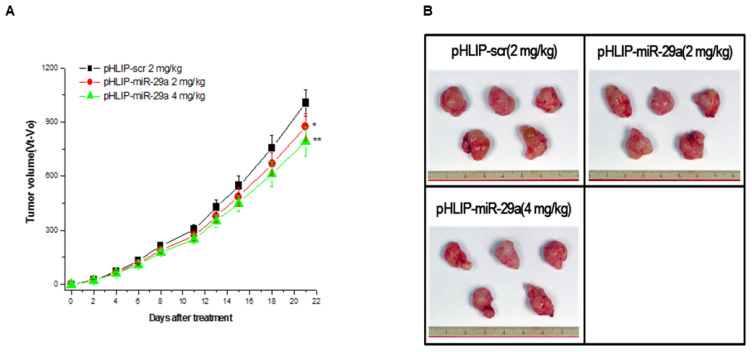
The administration of pHLIP-miR-29a inhibits pancreatic tumor progression in a pancreatic ductal adenocarcinoma-bearing mouse model. (**A**) CFPAC-1 tumor-bearing nude mice received intravenous injections of pHLIP-miR-29a, and tumor volumes were assessed at the indicated days following treatment (*n* = 5 mice per group). * *p* < 0.05; ** *p* < 0.01. (**B**) Representative tumor images were taken three weeks after the administration of pHLIP-miR-29a or pHLIP-scr at doses of 2 mg/kg or 4 mg/kg.

To evaluate the effects of pHLIP-miR-29a on pancreatic tumor development, tumor growth was measured every 2–3 days throughout the study. Tumor volumes in pHLIP-miR-29a-treated mice were significantly smaller than those in pHLIP-scr-treated control mice (Figure 4, Supplementary Table S1). At a dose of 2 mg/kg, the tumor size in pHLIP-miR-29a-treated mice was reduced by 13.0% of those in pHLIP-scr-treated mice (*p* < 0.05). At a dose of 4 mg/kg, the tumor size was reduced by 21.2% (*p* < 0.01). Additionally, pHLIP-miR-29a-treated mice exhibited no changes in body weight or clinical signs of distress for toxicity assessment (Supplementary Figure S1B).

## 4. Discussion

In this study, we demonstrated that the systemic administration of pHLIP-siCEACAM6 and pHLIP-miR-29a conjugates effectively delivered tumor-suppressive siCEACAM6 and miR-29a to PDAC cells by targeting the acidic tumor microenvironment, which resulted in significant antitumor efficacy in mice.

Among the CEACAM family of proteins, CEACAM5 is a widely recognized biomarker and a validated candidate for targeted therapies across various cancer types [22,23]. Recent studies have investigated CEACAM6 for its role in cancer development and progression and have shown its potential as a therapeutic target for several malignancies. Several studies have reported that CEACAM6 is overexpressed in numerous cancers, especially in NSCLC, colorectal carcinoma, and PDAC, and have explored various strategies targeting CEACAM6, including monoclonal antibodies, antibody–drug conjugates, chimeric antigen receptor (CAR) T-cells, and bispecific T cell engagers [3].

Previous studies targeting CEACAM6 have shown promising results in disrupting tumor cell adhesion, promoting apoptosis, and overcoming chemoresistance mechanisms in preclinical pancreatic cancer models. CEACAM5- and CEACAM6-targeted CAR T-cells show high antitumor efficacy in vitro and in animal models, highlighting their potential as effective immunotherapeutic agents [24]. Furthermore, multiple anti-CEACAM6 agents that exhibit favorable therapeutic effects in preclinical pancreatic adenocarcinoma models have been developed [17,25,26,27]. While these previously mentioned studies focused on antibody-based targeted therapies, RNA therapy is an emerging strategy in cancer treatment that utilizes RNA molecules to suppress oncogenes or enhance tumor-suppressive pathways. However, since RNA molecules are inherently unstable and prone to degradation, effective delivery systems are essential. This is the first study to introduce a novel therapeutic approach utilizing a pHLIP-mediated RNA delivery system to enhance the specificity and efficacy of CEACAM6-targeted therapy within an acidic tumor microenvironment in a PDAC model. The pHLIP can exhibit three distinct states: a water-soluble protein, a membrane-surface-bound protein, and an α-helical form embedded within the lipid bilayer. Under a physiological pH, pHLIP is primarily water-soluble, while exposure to slightly acidic conditions induces its insertion into membranes as an α-helix [28,29]. This unique characteristic makes pHLIP an attractive target for the selective labeling and tracking of acidic tissues in vivo. Since acidosis is a common feature of the tumor microenvironment [30], pHLIP specifically localizes to tumors. Therefore, the integration of pHLIP technology with CEACAM6-targeted agents presents a synergistic platform, offering the potential to significantly improve therapeutic outcomes in patients with CEACAM6-expressing PDAC.

The miR-29 family (miR-29a/b/c) regulates multiple signaling pathways involved in cancer progression and has been shown to inhibit CEACAM6 transcription [31,32,33]. Chen et al. demonstrated that CEACAM6 plays a direct role in the epithelial–mesenchymal transition, migration, invasion, and metastasis of pancreatic cancer cells. They also reported that miR-29a/b/c regulates CEACAM6 at the post-transcriptional level in pancreatic cancer [14]. Similarly, Han et al. reported that miR-29a inhibited the growth, migration, and invasion of lung adenocarcinoma cells by targeting CEACAM6 [33]. Additionally, Son et al. reported that miR-29a-based therapy inhibits tumor growth in a lung adenocarcinoma xenograft model [20]. Furthermore, Duxbury et al. investigated the effects of CEACAM6 gene silencing in PDAC cell lines. They reported that CEACAM6 knockdown reduced cellular invasiveness, decreased anoikis resistance, and suppressed metastatic potential in PDAC cell lines [15]. In another study, Duxbury et al. reported the therapeutic potential of siRNA targeting CEACAM6, which showed tumor regression in a PDAC xenograft model [34]. Similarly, Son et al. reported that CEACAM6 gene silencing-based therapy inhibited tumor growth in a lung adenocarcinoma xenograft model [21]. Therefore, both siCEACAM6 and miR-29a may act as tumor suppressors in PDAC by inhibiting CEACAM6 expression. Our findings are consistent with these results, demonstrating that siCEACAM6 and miR-29a exhibit significant antitumor effects in a PDAC model.

In our study, mice treated with pHLIP-siCEACAM6 or pHLIP-miR-29a exhibited no significant weight loss or clinical signs of toxicity, suggesting minimal systemic adverse effects. Ensuring the safety of RNA-based therapeutics is critical, particularly in systemic delivery approaches. The pHLIP technology used in this study was designed to selectively target the acidic tumor microenvironment, thereby minimizing unintended RNA uptake via normal tissues. However, further investigations are needed to better understand the effects of siCEACAM6 and miR-29a delivery on normal tissues and the tumor microenvironment.

This study involved several limitations. First, although CEACAM6 has been reported to be associated with epithelial–mesenchymal transition and anoikis resistance [3,14], we did not conduct additional analyses to determine whether the tumor reduction observed in our study was mediated through these mechanisms or other pathways. Second, while CEACAM6 is well known to contribute to chemoresistance in pancreatic cancer [5,14,35], we did not evaluate the potential synergistic effects of combining gemcitabine with siCEACAM6-pHLIP or miR-29a-pHLIP, which warrants further investigation to enhance the clinical relevance of CEACAM6-targeted RNA therapies. Third, we did not perform a histological analysis of normal tissues in pancreatic adenocarcinoma-bearing mice to assess potential off-target effects. Future studies should include comprehensive histopathological evaluations to further confirm the safety of this approach.

## 5. Conclusions

Our findings demonstrated the efficacy of the pHLIP-mediated delivery of siCEACAM6 and miR-29a as a promising therapeutic strategy for PDAC. The pHLIP technology, which targets the acidic tumor microenvironment, represents an innovative approach to the delivery of small RNAs to pancreatic cancer cells, offering significant potential for pancreatic cancer treatment.

## Data Availability

The data presented in this study are available upon request from the corresponding author.

## Supplementary Materials

The following supporting information can be downloaded at https://www.mdpi.com/article/10.3390/medicina61040598/s1: Figure S1: Body-weight changes in xenograft model for toxicity assessment following pHLIP-siCEACAM6 (A) and pHLIP-miR-29a (B) administration; Table S1. Changes in tumor size and final tumor weight.

## Abbreviations

PDAC	Pancreatic ductal adenocarcinoma
pHLIP	pH-low insertion peptide
siRNA	Small-interfering RNA
CEACAM6	Carcinoembryonic antigen-related cell adhesion molecule 6
