# Supplementary material for: Targeted Suppression of CEACAM6 via pHLIP-Delivered RNAs in Pancreatic Ductal Adenocarcinoma
Source: Medicina (Kaunas). 2025 Mar 26;61(4):598. doi: 10.3390/medicina61040598 (PMC12028928; doi:10.3390/medicina61040598)
Supplement: Supplementary file 1 [file medicina-61-00598-s001.zip › medicina-3484290-supplementary.pdf]

Targeted suppression of CEACAM6 via pHLIP-delivered RNAs in pancreatic ductal adenocarcinoma

Hongsik Kim[1†], Chang Gok Woo[2†], Seung-Myoung Son[2], Yong-Pyo Lee[1], Hee Kyung Kim[1], Yaewon Yang[1], Jihyun Kwon[1], Ki Hyeong Lee[1], Ho-Chang Lee[2], Ok-Jun Lee[2], Hye sook Han[1]

[1]Department of Internal Medicine, Chungbuk National University Hospital, Chungbuk National University College of Medicine, Cheongju, Republic of Korea

[2]Department of Pathology, Chungbuk National University Hospital, Chungbuk National University College of Medicine, Cheongju, Republic of Korea

Supplementary Information

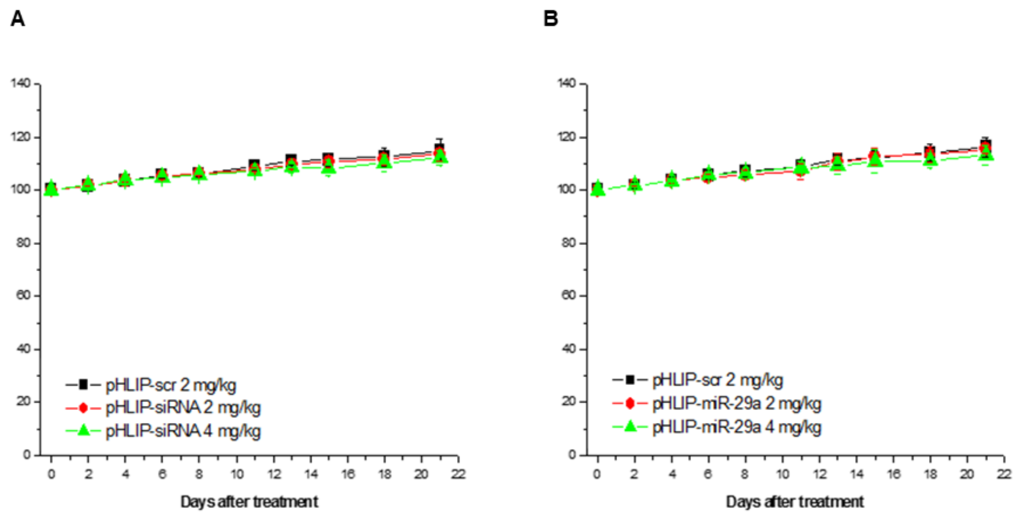

Supplementary Figure S1. Body weight changes in xenograft model for toxicity assessment following pHLIP-siCEACAM6 (A) and pHLIP-miR-29a (B) administration.

(a) Changes in body weight in experimental mice treated with pHLIP-siCEACAM6 during the treatment period (n = 5 mice/group). (b) Changes in body weight in experimental mice treated with pHLIP-miR-29a during the treatment period (n = 5 mice/group). Data are shown as mean ± s.d

Supplementary Table S1. Changes in tumor size and final tumor weight

Group (n=5)	Doses (mg/kg)	Tumor volume(*Vt-Vo*)†										Tumor weight (mg)
		day 0	2	4	6	8	11	13	15	18	21	22
pHLIP-scr (for mR-29a)	2	0.0 ± 0.0	23.9 ± 2.9	69.9 ± 9.3	128.6 ± 13.7	213.3 ± 18.2	303.7 ± 26.1	426.7 ± 42.8	546.4 ± 53.9	756.3 ± 69.4	1005.7 ± 73.4	1189.9 ± 120.5
pHLIP-miR -29a	2	0.0 ± 0.0	22.4 ± 2.7	64.1 ± 3.9	115.6 ± 9.5	189.6 ± 16.9	269.1 ± 26.3	377.5 ± 33.7	484.4 ± 51.3	669.0 ± 60.7	874.5 ± 73.4*	1013.5 ± 84.3*
			6.4%	8.2%	10.1%	11.1%	11.4%	11.5%	11.3%	11.5%	13.0%	14.8%
	4	0.0 ± 0.0	21.4 ± 0.8	61.6 ± 6.1	110.3 ± 10.6*	177.6 ± 17.1*	252.3 ± 23.9*	352.3 ± 33.4*	449.4 ± 42.9*	612.7 ± 71.7*	792.8 ± 80.6**	916.9 ± 88.5**
			10.5%	11.8%	14.2%	16.7%	16.9%	17.4%	17.8%	19.0%	21.2%	22.9%
pHLIP-scr (for siRNA)	2	0.0 ± 0.0	22.6 ± 2.2	71.3 ± 7.7	129.1 ± 9.8	214.0 ± 15.0	307.5 ± 30.3	429.6 ± 41.7	551.0 ± 56.5	761.1 ± 69.7	1027.9 ± 74.1	1228.2 ± 121.0
pHLIP-siRNA	2	0.0 ± 0.0	20.9 ± 3.1	64.9 ± 4.8	115.7 ± 9.3	187.6 ± 21.9	268.4 ± 28.5	374.3 ± 35.2	480.9 ± 39.2	658.8 ± 74.4	879.0 ± 71.3*	1022.9 ± 83.6*
			7.7%	9.0%	10.4%	12.3%	12.7%	12.9%	12.7%	13.4%	14.5%	16.7%
	4	0.0 ± 0.0	19.9 ± 2.2	62.2 ± 3.7*	108.0 ± 10.8*	177.2 ± 19.2**	251.0 ± 24.0*	346.5 ± 39.9*	441.2 ± 51.6*	597.6 ± 80.1**	769.6 ± 88.2**	903.6 ± 95.0**
			11.8%	12.8%	16.4%	17.2%	18.4%	19.3%	19.9%	21.5%	25.1%	26.4%

Significant figures (t-TEST) : * $p<0.05$, ** $p<0.01$, *** $p<0.001$(vs Vehicle only, pHLIP-scr for mR-29a, for siRNA)

† *$\Delta t = Vt-Vo$, Vt(Measurement of the tumor volume), Vo(Initial tumor volume)*

‡ Inhibition Rate (%, vs Vehicle only, pHLIP-scr for mR-29a, for siRNA)
